# Revolutionizing Healthcare With Paper‐Based Nucleic Acid Testing

**DOI:** 10.1002/EXP.20240128

**Published:** 2026-05-28

**Authors:** Hong Zhang, Yi Yang, Heng Li, Xiaolin Hu, Hui Xiao, Dou Wang, Sergio Benardini, Wei Gu, Yang Luo

**Affiliations:** ^1^ Department of Laboratory Medicine, Chongqing Center for Clinical Laboratory Chongqing Academy of Medical Sciences Chongqing General Hospital School of Medicine Chongqing University Chongqing P. R. China; ^2^ Department of Laboratory Medicine, West China Second University Hospital, and Key Laboratory of Obstetric & Gynecologic and Pediatric Diseases and Birth Defects of Ministry of Education Sichuan University Chengdu P. R. China; ^3^ College of Life Science and Laboratory Medicine Kunming Medical University Kunming Yunnan P. R. China; ^4^ Guangdong Provincial Key Laboratory of Advanced Biomaterials Department of Biomedical Engineering Southern University of Science and Technology Shenzhen Guangdong P. R. China; ^5^ Department of Experimental Medicine University of Tor Vergata Rome Italy

**Keywords:** microfabrication, nanomaterial, nucleic acid amplification techniques, paper chromatography, point‐of‐care testing

## Abstract

The rapid and accurate identification of nucleic acids (NAs) is essential to early diagnosis and treatment of disease in healthcare. Paper‐based analytical devices (PADs) offer a preferred alternative to laborious, expensive, and time‐consuming conventional nucleic acid testing (NAT) methods with considerable success. However, their development has been constrained by challenges in NA recognition and signal amplification within a restricted paper fiber network. Recently developed PADs, which establish a three‐dimensional environment for NA reactions by the integration of porous fibers and modified substrate materials, are limited by the precision of base identification and user convenience. In this review, we focus on paper‐based analytical devices with enhanced point‐of‐care applicability for NA detection and discuss the strategies for enhanced NA cascade signal amplification and background noise suppression. Emphasis is placed on the integrated detection of NA molecules from input to output, highlighting the effective signal amplification and rapid signal output of low‐concentration NA molecules of the paper‐based assay in complicated detection systems.

## Graphical abstract

This work summarizes strategies to enhance paper‐based devices for nucleic acid testing. Key approaches include optimizing paper platforms, improving nucleic acid amplification, and refining labeling/signaling methods. These advancements aim to boost sensitivity, speed, and usability, making paper‐based diagnostics more effective for point‐of‐care applications.

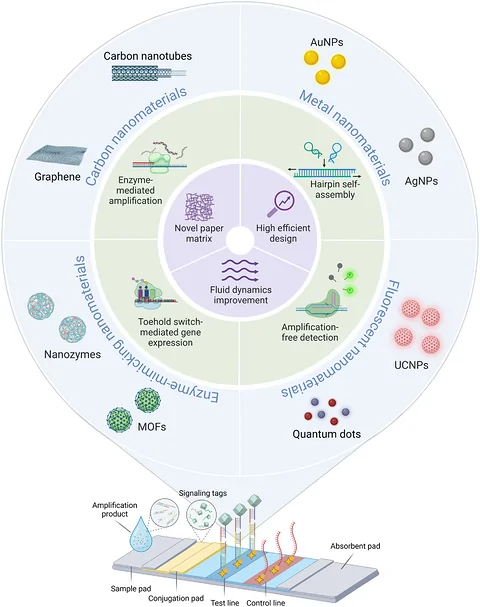

## 1 Introduction

The escalating emergence of novel infectious diseases and heightened public health consciousness have led to a surge in demand for point‐of‐care testing (POCT) solutions that facilitate self‐testing at home and expedite hospital diagnostics [1]. Since the pioneering work of Whiteside's research group in 2007 [2], which established paper‐based microfluidics as a viable analytical platform, there has been a significant upsurge in the development of paper‐based analytical devices (PADs) for healthcare applications (Figure 1). These devices are particularly appealing due to their portability, disposability, and cost‐effectiveness. Starting with basic qualitative tests for proteins and progressing to today's quantitative analysis of nucleic acids (NAs) and other substances, efforts are being made to develop compact and distributed PADs suitable for field applications to meet the growing demand for personal healthcare [3].

**FIGURE 1:**
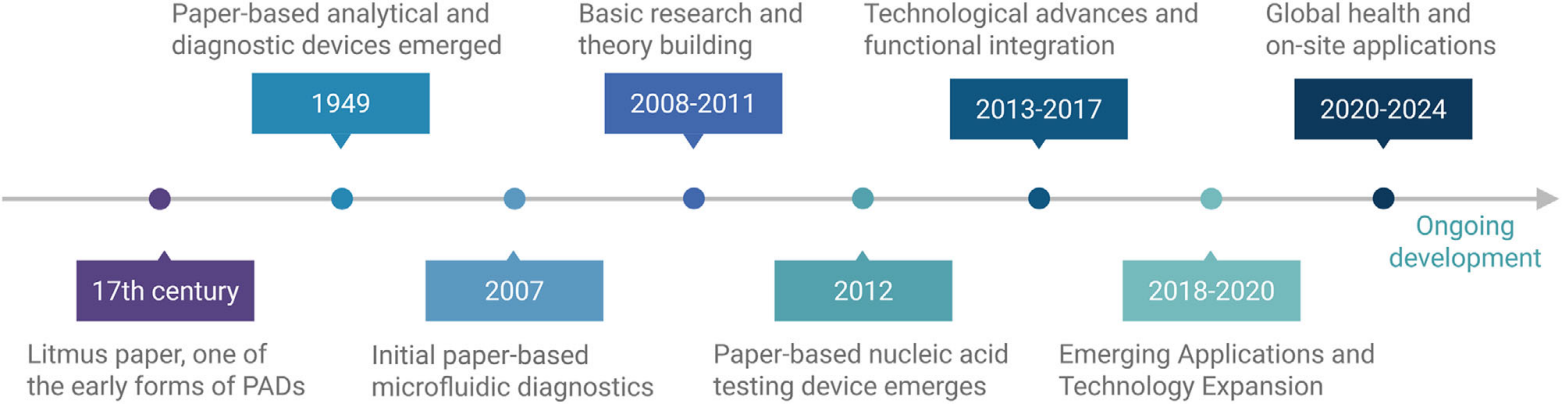
Timeline of major technological advances in paper‐based sensing. The evolution of paper‐based analytical devices began with Robert Boyle's 17th‐century discovery of acid‐base chromogenic properties in litmus extract, leading to litmus paper as an early paper‐based sensor. Müller et al. (1949) subsequently engineered paraffin‐treated filter paper for chromatography, establishing foundational diagnostic platforms [4]. In 2007, George Whitesides et al. introduced paper‐based microfluidic chips [2], catalyzing systematic theoretical studies (2008–2011) [5, 6, 7]. Subsequent advancements included nucleic acid testing platforms (2012) and multidimensional devices (2013–2017), achieving functional integration via engineered fluidics [8, 9, 10, 11]. By 2018, paper‐based diagnostics began to be used in more health applications and integrated with mobile devices such as smartphones, increasing the portability and accessibility of testing [12, 13, 14, 15]. Recent global public health demands have accelerated research in this domain (2020–2024), with intensified focus on refining on‐site analytical workflows to address point‐of‐care diagnostic requirements [16, 17, 18].

During the COVID‐19 outbreak, lateral flow assays (LFAs) and PADs for daily mass testing of SARS‐CoV‐2 infection [19] were been widely employed. Additional applications of these devices encompass cancer diagnostics, various viral infections, and allergy‐related microbial disorders [20]. Paper‐based substrates are manageable and suitable for modification, with their three‐dimensional pores offering an ideal platform for rapid extraction, amplification, and direct signal output in the detection of NA molecules [21]. Recent advancements in molecular biology techniques, particularly molecular amplification and gene editing methods, have mostly accelerated progress in paper‐based nucleic acid testing (NAT) [22]. Driven primarily by the need to address clinical diagnostics in point‐of‐care (POC) settings, the market for POC diagnostics on paper platforms was valued at USD 25.3 billion by 2022 and is expected to expand over the next ten years [23, 24]. This underscores the critical need for continuous innovation and clinical applicability, as further discussed in this review.

Despite the advancements in rapid NA detection using paper‐based sensors, challenges remain regarding sensitivity and stability, a limited dynamic range, and interferences in complex biological samples [25]. These limitations are frequently linked to the inherent characteristics of paper, including its porosity, which can unintentionally entrap NA strands and nanoparticle‐based probes within the cellulose fibers, resulting in false‐positive outcomes [26]. Moreover, integrating complex biochemical reactions into a paper platform presents challenges in terms of reagent stability and reliable design of detection platforms, which in turn impact the overall performance of PADs under field conditions [27]. To overcome the gap between PADs and conventional laboratory methods, research has focused on incorporating novel materials and structural designs, enzymatic amplification strategies, and integration with electronic devices [28, 29].

In this review, we concentrate on strategies to enhance the sensitivity of PADs for NAT, guided by three primary objectives (Figure 2). The review emphasizes methods to improve the performance of PADs, including the introduction of unique flexible fibers, surface modification techniques, and advancements in device engineering. Then, we summarize the application of various technological innovations in NA amplification and signal labeling in paper‐based assays, proposing practical improvements from different perspectives to effectively minimize background noise. Moreover, we discuss the innovative combination of amplification‐free NA detection and digital technologies with PAD, as well as prospective advancements. Finally, the review specifically introduces the concept of minimizing background noise in NA detection on test strips, emphasizing the cascade signal amplification of trace NA molecules within a three‐dimensional fiber space compared to prior reviews [30, 31, 32].

**FIGURE 2:**
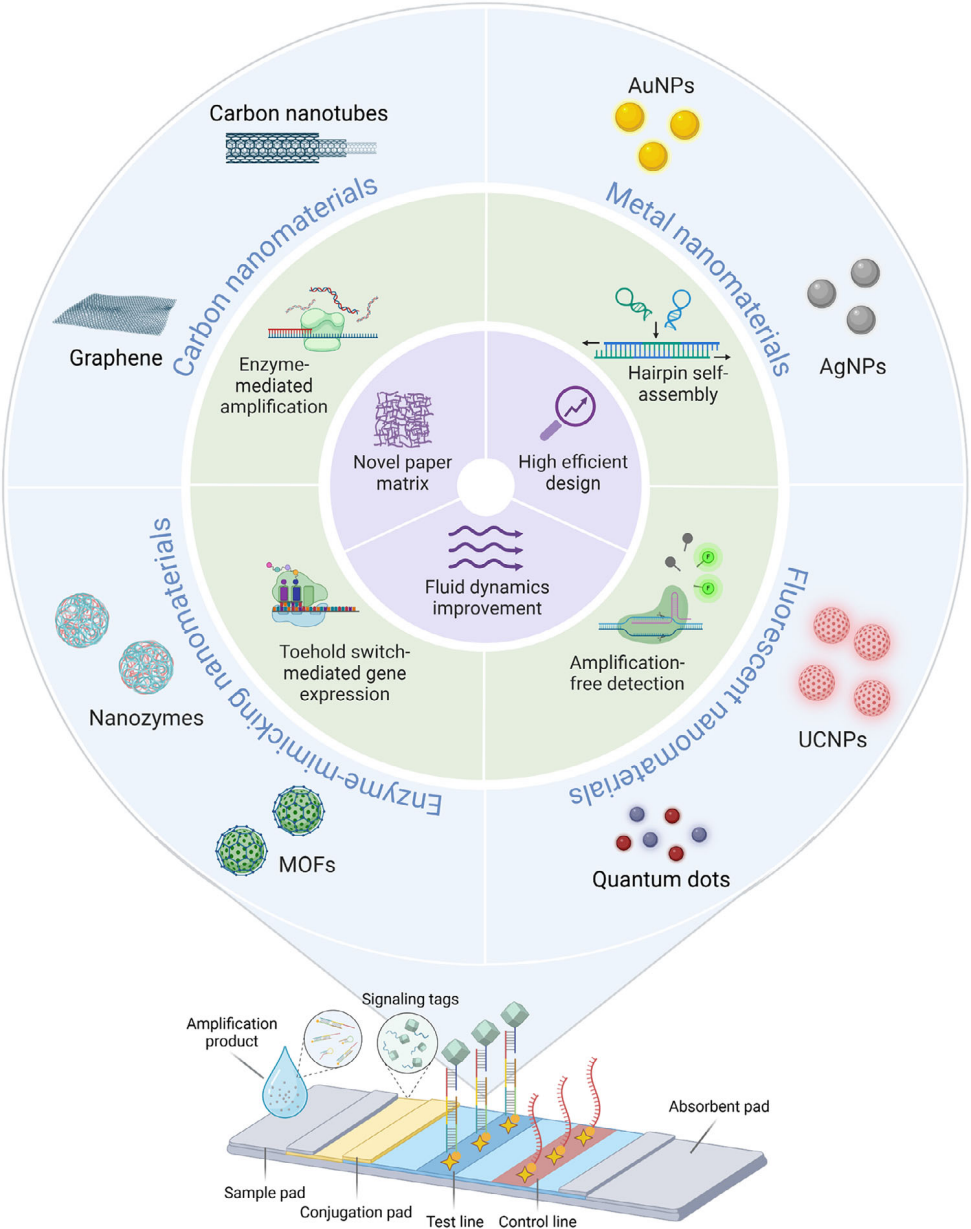
Overview of improving the capabilities of PADs for NAT. Strategies involve paper‐based platforms (purple), NA amplification (green), labeling, and signaling (blue) components.

## 2 Structure Design and Optimization of PADs

The PAD that hosts the NA reaction is not merely a static component, but it plays an active role in every step of the diagnostic process. It exerts an influence on fluid dynamics, reagent distribution, and reaction efficiency. Therefore, strategic design and optimization of PADs are essential to improving the diagnostic capability and efficiency of NAT.

### 2.1 Typical Structures of PADs

PADs have emerged as a promising platform for NAT due to their simplicity, cost‐effectiveness, and portability. These devices leverage the inherent properties of paper, such as capillary action and porous structure, to facilitate fluid transport and reaction kinetics. The structural design of PADs can be broadly categorized into two‐dimensional (2D) and three‐dimensional (3D) architectures. The 2D PADs, including LFAs, often comprise a singular plane of strips with patterned hydrophobic barriers that delineate the microfluidic channels and reaction zones, exemplifying the most basic and frequently investigated designs of paper‐based assays [33]. The precise control of liquid flow in 2D PADs can be achieved by imprinting soluble barriers within the plane of the paper. For instance, the researchers introduced a technique to control capillary flow on paper by imprinting roadblocks on the flow path with water‐insoluble ink and using the gradual formation of a void between wetted paper and a sheath of polymer tape to create timers [34]. Timers are positioned at key nodes to maintain capillary flow for a specified duration, facilitating the introduction of multiple liquids into multistep chemical reactions according to a programmed sequence (Figure 3A). These techniques enable 2D PADs to autonomously execute multistep chemical processes, including NA adsorption, washing, and the addition of amplification reagents, thereby improving the scalability of the detection. The assays utilize capillary action to facilitate sample flow through the device, enabling interaction with immobilized reagents to generate a visual readout. Tao et al. employed wax printing to establish a hydrophobic barrier on a two‐dimensional unmodified cellulosic substrate, allowing the division of sample loading and straight test zones [35]. Pre‐incubating the DNA dye SYBR Green I in the sample loading zone and adding amplified and purified HPV genomic samples exploited the unique interfacial interaction between SYBR Green I and unmodified cellulose. This method allowed concentration‐dependent elution of SYBR Green I into the straight test zone by reaction amplicons, allowing the 2D PAD to quantify amplification products by measuring fluorescence distance. Despite the advantages of 2D PADs, their planar architecture limits their ability to handle complex samples and restricts the detection throughput.

**FIGURE 3:**
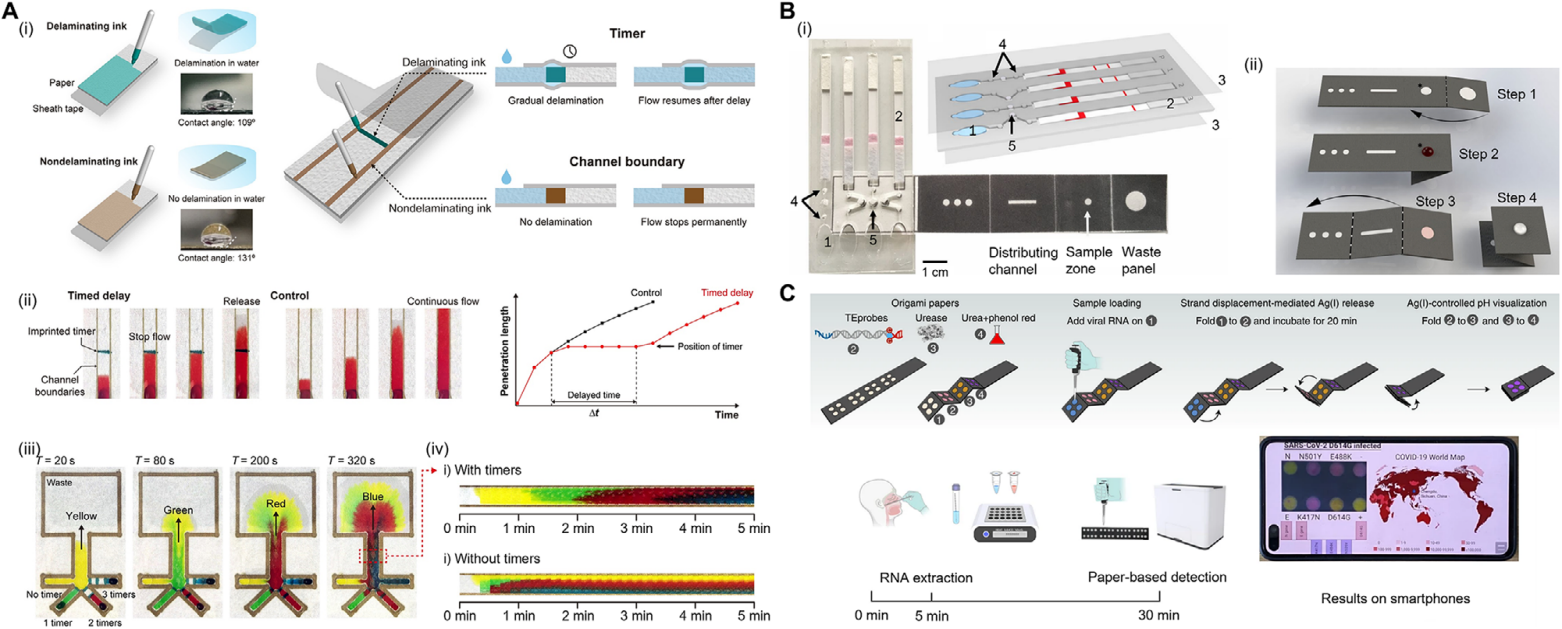
Typical structures of PADs and applications. (A) 2D PADs for capillary flow control via sheath tape delamination. Reproduced with permission [34]. Copyright 2021, The American Association for the Advancement of Science. (i) Manipulation of capillary flow on paper by imprinting patterns to modulate the paper‐tape adhesion. (ii) Time‐lapse images of a dye solution progressing on paper with a timer (left) and without a timer (right). (iii) Time‐lapse images of a test paper platform with four dye solutions simultaneously introduced from different inlets. (iv) Change of observed color at the middle of the shared channel as a function of time with (top) and without timers (bottom). (B) Folded paper‐based microfluidic device that enables LAMP‐based multiplexed detection of malaria in blood. Reproduced under terms of the Creative Commons Attribution 4.0 International License [36]. Copyright 2019, Julien Reboud. (i) The numbers represent the primary functional areas of the device, specifically, 1, buffer chambers (as a finger pump); 2, lateral flow DNA detection strip; 3, acetate films; 4, filter paper‐based valves; and 5, filter paper (for LAMP reaction). (ii) The diagram shows in detail how this strip is folded. (C) 3D PADs for the colorimetric detection of SARS‐CoV‐2 variants at single‐nucleotide resolution. Reproduced with permission [37]. Copyright 2022, Springer Nature.

To overcome the limitations imposed by the 2D structure, particularly regarding the handling of complex samples and detection capacity, 3D PADs have been conceived. These are created by layering, folding, or stacking multiple 2D layers, which enables the incorporation of various reaction chambers, fluidic channels, and detection areas in an integrated device. The additional dimension enables the execution of complex, multi‐step assays, including sample preparation, NA amplification, and detection, within a single unit. An example is a folding analysis technique for rapid malaria analysis [36]. By folding the paper into different functional areas, the sample can be guided through the various testing stages, from sample preparation to readout, without needing to be transferred between different instruments (Figure 3B). This design seamlessly integrates the NA extraction and amplification processes into a single device, allowing for the detection of multiple pathogens in a single sample addition step and eliminating the loss of NA and contamination associated with multi‐step pipetting. The 3D configuration also helps in reducing cross‐contamination between different reagents and allows for more intricate fluidic networks and enhanced reaction dynamics through the stacked layers [38]. Additionally, the 3D design enables the integration of various detection methods, including colorimetric, fluorescent, and electrochemical assays, providing a versatile platform for NAT [39]. In another study, Zhang et al. developed an innovative multifunctional detection platform termed MARVE (standing for multiplexed, NA‐amplification‐free, single‐nucleotide‐resolved viral evolution), which was ingeniously integrated into foldable PADs [37, 38]. Leveraging the multi‐compartmental functionality of PADs, they were able to independently perform NA strand displacement reactions, precisely capture the thermodynamic energy loss associated with single‐base pair mismatches, and amplify the recognition of viral RNA through metal ion‐controlled urease cleavage reactions (Figure 3C). These reactions were ultimately read out by a smartphone‐based colorimetric assay, enabling rapid detection of viral variants. In a test cohort of 50 samples, MARVE demonstrated 100% specificity, attesting to its efficiency and accuracy in detecting viral variants.

### 2.2 Specific Optimization of PADs for NAT

The paper matrix is central to PAD, actively influencing fluid dynamics and chemical reactions, not merely providing structural support. Cellulose and fiberglass papers are commonly used due to their absorbency and permeability, but they suffer from limited mechanical strength and inadequate chemical properties, which can hinder assay sensitivity and precision [26, 40, 41, 42]. A comprehensive comparison of the merits and drawbacks of various paper matrices for NAT is delineated in Table 1.

**TABLE 1:** Analysis of the characteristics of several paper substrates.

Type	Advantages	Disadvantages	Materials and characteristics	References
Cellulose paper	Low‐cost; high hydrophilicity and porosity; fast fluid flow rates; easy to modify.	Uneven pore size and distribution may affect sample distribution; low mechanical strength after wetting; limited capacity for NA adsorption.	Fusion 5 filter paper: rapid NA extraction (a few minutes), but low NA purity.	[43]
			FTA card: time‐consuming (≈25 min), but better NA extraction.	[26, 44]
Fiberglass paper	Enhanced mechanical strength; good chemical stability; heat‐resistant.	Increased cost; possible bioirritation.	Glass fiber.	[45, 46, 47]
Coated paper	Multi‐functional coatings; flexible detection applications.	High‐cost; complicated preparation; high demand for preservation and transportation conditions.	Carbon nanotubes: multi‐active binding sites, and rapid current response.	[48, 49]
			Polyvinyl alcohol: automated fluid transfer.	[34, 50]
			Graphene oxide (GO): anti‐pollution and reduced background fluorescence interference.	[51]
Nanofiber paper	Enhanced NA adsorption and retention; rapid fluid transfer; customized functions; high mechanical strength.	Requires higher manufacturing technology; high‐cost; batch‐to‐batch variation.	Nanofibrillated cellulose: remarkable mechanical properties.	[52, 53, 54]
			Carbon nanofibers: excellent filtration and electrochemical properties.	[55]
			Electrospun fibres: better binding specificity, easy to modify, and adjustable flow rate.	[56, 57]
Composite paper	Improved chemical stability and mechanical strength; multi‐functional.	Complicated preparation; high‐cost; potential for environmental contamination; difficult to recycle.	Nitrocellulose membrane doped with cellulose nanofibers: improved biomolecular adsorption capacity, and enhanced sensitivity.	[58]
			Multi‐modified fibers: relatively low‐cost and high NA adsorption specificity.	[59]

Recent advancements in flexible fiber manufacturing and modification technologies have greatly impacted the paper matrix field, particularly in the creation of nanofiber paper [60]. This nanoscale fiber‐based paper features a large specific surface area and ultrafine pores (10–50 nm) that enhance both the adsorption of NAs and the immobilization of biomolecules [61, 62], which is crucial for initiating rapid downstream amplification and reducing non‐specific primer binding [63]. Furthermore, thermal conductivity is another key for stable temperature maintenance during NA amplification on PADs. Li et al. developed a nanofiber paper with exceptional thermal conductivity through the incorporation of silver nanoparticles (AgNPs). This material showed a thermal conductivity efficiency 5.35 times higher than that of conventional nanofiber paper [52]. The improved thermal conductivity accelerates heat transfer between the heating device, paper, and NA molecules during isothermal amplification, thereby augmenting both amplification efficiency and detection performance. The coated paper involves the application of specialized substances, like polymers or nanostructures, onto the paper surface. For instance, Niharika Gupta et al. developed a conductive coated paper by using carboxylated multiwalled carbon nanotubes (cMWCNTs) as a conductive ink on a paper substrate for detecting *Neisseria gonorrhoeae* [48]. The high surface activity and enhanced electron transfer ability of cMWCNTs expanded the linear dynamic range by four orders of magnitude (100 fM–100 nM) compared to plain graphite‐based designs [64]. Moreover, the application of organic polymerization coatings, such as chitosan, 1,4‐phenylenediisothiocyanate, and polyethyleneimine, results in the formation of a dense layer of positive charge on the paper's surface. The addition of these coating layers enhances the ability to bind and retain NA molecules through electrostatic forces, thereby increasing the effectiveness of NA amplification and detection [43, 65, 66]. The composite paper integrates functional polymers or nanostructures into fibers during manufacturing [67, 68]. This integration, such as the addition of cellulose nanofibers into nitrocellulose membranes, modifies the paper's porosity, surface layer, and total surface area, enhancing its biomolecule binding capacity, which in turn boosts the sensitivity for detecting *Staphylococcus aureus* by twenty times [58].

Another aspect of enhancing PAD performance is to optimize hydrodynamic properties. One significant drawback of PADs is their reduced wicking speed, leading to slower fluidic response times and a higher likelihood of sample contamination, particularly when multi‐targets are being tested [69]. Innovations have introduced a gap or channel between closely spaced paper sheets, boosting flow rates by at least 169 times compared to single‐layer PADs [70]. However, this phenomenon appears to contradict the Lucas–Washburn equation (Equation (1)), which is used to model fluid transport in porous materials. Researchers have since used experimental and analytical modeling to explain this flow enhancement [71]. Laplace pressure, the pressure differential at a liquid/gas interface, plays a crucial role in multilayer PADs. As illustrated in Equation (2), as liquid enters the device, it forms a liquid/gas interface between paper layers, increasing Laplace pressure with the liquid/gas interface area, which is proportional to the gap height. Furthermore, by adjusting the pore size of the paper, surface treatment, or the properties of the fluid (such as viscosity), the energy lost due to friction between fluid molecules within the multilayer PAD can be minimized, resulting in reduced viscous dissipation. The combined effect of increased Laplace pressure and decreased viscous dissipation accelerates fluid flow within the interlayer gaps, thereby enhancing the rapid detection capability of PADs. Based on Equation (2), Robert et al. demonstrated that fluid flow rate in PADs increases exponentially with gap height, reaching about 240 times the velocity of a single‐ply device at a maximum gap height of 390 µm. Weight, centrifugal force, pressure differential, and surface imperfection have been used to accelerate liquid in other studies [72, 73, 74]. These investigations have enhanced the efficiency and performance of PADs in time‐sensitive manipulations and measurements.

(1) $$l\;\left(t\right)=\sqrt{\frac{\gamma r^{\prime }t\cos\theta }{2\mu }}\;$$

where *l(t)* is the distance traversed down the channel (m) at time *t* (s), *γ* is the interfacial tension (N·m^−1^), *r’* is the mean capillary radius (m), *θ* is the fluid contact angle on the paper, and *µ* is the fluid viscosity (N·s·m^−1^)

(2) $$t=\frac{c}{2a+1}\;\left[\frac{1}{u^{2}}-\frac{1}{u_{0}^{2}}{\left(\frac{u_{0}}{u}\right)}^{1/\left(a+1\right)}\right]$$

where *a* = 3*t*2 h/ *h* ^2^ and *c* = *γt*2 h cos^2^ *θ* / *µh*. This nonlinear differential equation is a form of the d'Alembert equation and was originally solved by Jacob Bernoulli, where *µ* is the fluid viscosity (N·s·m^−1^), Laboratory results for *u_0_* may then be used as input to the model.

It is noteworthy that the fluid flow depends on the capillary action of the remaining unwetted portion of the paper. Once the paper matrix reaches its absorbable limit, sample movement may be impeded by limited space, posing a challenge for subsequent washing processes. An effective mitigation strategy is to elongate the channel or employ a sacrificial waste pad to maintain a consistent flow during the washing process, thereby preserving the portability of the device, albeit needing periodic manual intervention [33, 75]. Innovative washing techniques, such as adopting three‐dimensional washing designs and strategically applying active solutions, have proven effective in removing residual reaction inhibitors and unreacted NA probes [76, 77, 78]. These innovative techniques address the issue of non‐specific adsorption due to the porous nature of the paper matrix by leveraging elements like gravity and fluid evaporation to modulate the hydrodynamic characteristics within the paper. They are essential for reducing background noise and facilitating NA amplification reactions in downstream steps.

## 3 Molecular Amplification Strategies for Paper‐Based NAT

Molecular amplification strategies play a pivotal role in NAT as they substantially enhance the relative concentration of NAs in the analytical system. This enhancement is vital for strengthening the effectiveness of subsequent steps, including NA labeling and signaling, thereby significantly improving the accuracy and fidelity of diagnostic and analytical outcomes.

### 3.1 Enzyme‐Mediated Amplification

Common enzyme‐mediated amplification techniques used in PADs typically include loop‐mediated isothermal amplification (LAMP) [79], NA sequence‐based amplification (NASBA) [80], recombinase polymerase amplification (RPA) [81], and strand displacement amplification [82]. LAMP and RPA are most commonly used in large‐scale screening and resource‐limited environments due to their speed, efficiency, and versatility in handling templates.

LAMP achieves exponential amplification of target DNA sequences using a specialized primer set and a strand‐displacing polymerase, generating up to 10^9^ copies within an hour, a rate notably higher than PCR [83]. This efficiency makes LAMP particularly suitable for use in PADs. Connelly and colleagues introduced a “paper machine” PAD that utilized LAMP for NA detection of *Escherichia coli*, achieving a detection limit of just five cells [84]. The device integrated essential steps‐sample application, buffer washing, and amplification into a compact, disposable sliding‐stripe setup, with results visible via a smartphone or portable UV light. However, it still required external equipment for result analysis and an incubator for heating. Addressing this limitation, Choi et al. developed a battery‐powered, handheld unit to replace the incubator, integrating LFA and LAMP into a portable, cost‐effective PAD able to detect as few as 3 × 10^3^ DNA copies [85]. Furthermore, the LAMP's capacity for visual detection through colorimetric changes is especially advantageous for paper‐based formats, such as observing colorimetric changes induced by Mg^2+^‐sensitive dyes or pH indicators that react to by‐products like pyrophosphate, which allows endpoint determination of the amplification process, with sensitivity up to a single copy per milliliter [86, 87]. The high amplification efficiency of LAMP reactions comes with the challenge of enduring high temperatures, which can affect the paper matrix and incorporated functional elements, such as paraffin waxes for hydrophobic channels, paper substrate strength, and indicators for LAMP reaction. Recently, Daehan Nam et al. proposed a method for efficient LAMP amplification at the physiological temperature of 37°C [88], enhancing low‐ temperature performance by fine‐tuning MgSO_4_, deoxynucleoside triphosphates (dNTPs), and DNA probe lengths. However, a loss of sensitivity was observed during ligation, indicating a need to increase ligation efficiency. Building on this, Cai and co‐workers developed phosphorothioated primers‐LAMP (PS‐LAMP), which employs PS to facilitate hairpin formation and extension at concatemer termini, enabling operation at lower temperatures [89]. With the addition of urea and single‐stranded binding protein, PS‐LAMP's sensitivity and specificity at 40°C matched those of a standard LAMP reaction at 65°C. However, further research is required to validate the feasibility of this approach in PADs for NAT and fully utilize the potential of LAMP in low‐resource and POC diagnostic applications.

RPA offers faster reactions than LAMP, completing in just 10–15 min and operating efficiently at lower temperatures (37°C–42°C), which facilitates the miniaturization of power‐dependent devices for RPA‐based detection [90]. Tsaloglou et al. developed a portable, paper‐based platform with a mobile electrochemical detector for electrochemical analysis of *Mycobacterium tuberculosis* [91]. The signal conversion was facilitated by electroactive, sequence‐specific probes, and automated detection was achieved by inserting a preloaded RPA test strip into a heating module after sample addition. This is mainly due to the stability of the RPA dry reagent, which makes transportation and storage more convenient. Upon rehydration, these reagents maintain assay efficiency comparable to solution‐based RPA, with a 90% sensitivity compared to RT‐PCR [92]. The specific high‐sensitivity enzymatic reporter unlock (SHERLOCK) assay, which utilizes RPA in PADs for NA analysis, has proven effective in detecting a wide range of infections and genetic markers [93] (Figure 4A). This platform combines RPA's high amplification potency with T7 transcription and LwCas13a‐mediated RNA cleavage to achieve ultrasensitive detection of single‐stranded DNA (ssDNA) targets at concentrations as low as 2 aM, rivaling the sensitivity of digital droplet PCR and quantitative PCR [94]. More recently, Tang et al. introduced CLIPON (CRISPR and Large DNA assembly Induced Pregnancy strips for signal‐ON detection), a method for universal target signal transformation using commercial pregnancy test strips (Figure 4B). CLIPON integrates commercial PTS with four biological components, like human chorionic gonadotropin (hCG), CRISPR‐Cas12a, crRNA, and cauliflower‐like large‐sized DNA assemblies (CLD). The CLD's spatial blocking effect enables the Cas12a/crRNA complex to release CLD‐bound hCG upon target identification, generating a colorimetric signal on the PTS [95]. The main advantage of this design is the use of readily available commercial PTS for signal outputs, offering a simple and versatile biosensing strategy for POC or point‐of‐need detection, especially in resource‐limited or large‐scale detection scenarios. However, the widespread application of RPA is hindered by several factors, including the proprietary nature of the underlying technology and its associated high costs. The complex composition of enzymes and additives required often necessitates commercial kits, primarily from TwistDx, which can delay deployment during time‐sensitive situations like infectious disease outbreaks. It is important to recognize that simplifying the experimental protocol by reducing the reaction temperature can lead to a significant increase in the number of non‐specific amplification products. These products are a major source of background noise in isothermal NA amplification, which complicates data interpretation [96, 97]. Recent studies have demonstrated that the incorporation of certain additives into amplification systems can significantly enhance the specificity of the amplification reaction. For instance, Jiang et al. found that the non‐polar characteristics of tetramethylammonium chloride (TMAC) contribute to the stabilization of DNA or RNA structures and increase the melting temperature of NA strands [98]. This stabilization makes it more difficult for the NA duplexes to denature at lower temperatures, thereby reducing the likelihood of non‐specific binding and potential mismatches during amplification. TMAC's inhibitory effect on non‐specific amplification is concentration‐dependent, with a 60 mM concentration capable of eliminating such amplification. Similarly, Gao et al. reported that pullulan (PLL), a linear polysaccharide polymer, can stabilize multiple primers in the NA amplification system, reducing non‐specific binding and amplification through physical or steric hindrance [99]. Moreover, the inhibitory effect of PLL on non‐specific amplification is concentration‐dependent. As the concentration of PLL increases, the suppression of non‐specific amplification becomes more pronounced, indicating that PLL may be more effective at stabilizing primers or influencing the activity of DNA polymerase at higher concentrations. Graphene oxide (GO) can functionalize paper matrices and selectively bind to single‐stranded NAs (ssDNA and RNA) through π‐stacking interactions and hydrogen bonding [100, 101]. During amplification, high primer concentrations can lead to primer dimers and mismatched hybrids, causing nonspecific amplification and background fluorescence signals. GO can adsorb these primers, which are then released upon target NA addition due to stronger base‐pairing affinity, inhibiting nonspecific hybridization in the absence of the target [102]. GO also quenches background fluorescence from excess DNA staining dyes, improving the signal‐to‐noise ratio, and adsorbs excess *Bst* DNA polymerase, suppressing nonspecific amplification caused by polymerase excess [102, 103].

**FIGURE 4:**
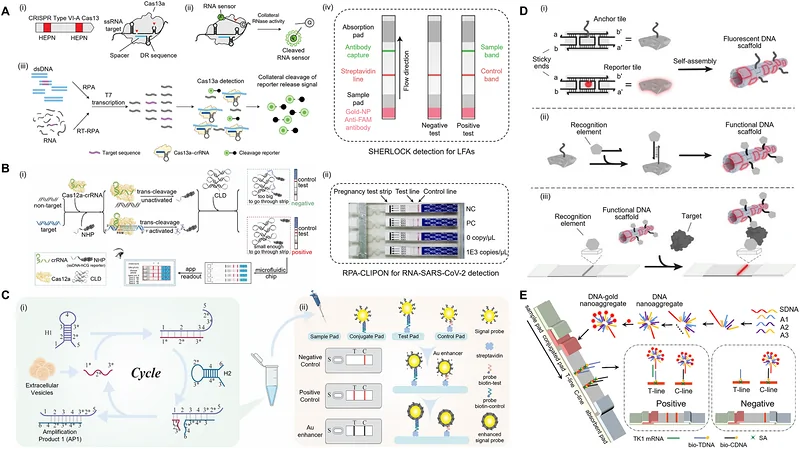
Application of NA amplification techniques in PADs. (A) Cas13 complex collateral activity and SHERLOCK assay. Reproduced with permission [93]. Copyright 2019, Springer Nature. (i) CRISPR‐Cas13 RNA targeting complex components. (ii) Reporter unlocking via CRISPR−Cas13 collateral RNase activity. (iii) SHERLOCK detection assay and (iv) single‐plex colorimetric lateral flow reaction. (B) Conversion of commercial pregnancy test strips to general POCT devices for NAT. Reproduced with permission [95]. Copyright 2022, Wiley‐VCH GmbH. (i) CLIPON using the detection of a double‐stranded DNA target as the target of interest. (ii) A microfluidic chip for pregnancy test strips to detect negative and positive specimens for SARS‐CoV‐2 pseudovirus. (C) The principle of the CHA strip for mRNA detection. Reproduced under the terms of the Creative Commons Attribution 4.0 International License [104]. Copyright 2021, Hongxia Li. (i) CHA cycle for target amplification. (ii) Gold enhancement technique for LFA of glypican‐1 mRNA. (D) DNA‐based scaffolds for a lateral flow test. Reproduced with permission [105]. Copyright 2023, Wiley‐VCH GmbH. (i) DNA tiles assembled through the hybridization of five different DNA strands displaying four sticky ends (a, b, a’, b’) each of five nucleotides. (ii) Adding a modified DNA strand complementary to the anchor domain makes it possible to decorate the DNA scaffold with different recognition elements. (iii) Scheme of a sandwich format of an LFA that employs functional DNA scaffolds as both reporter tags and recognition elements. (E) Preparation of DNA‐gold nanoaggregate and visual detection of TK1 mRNA by NALFA. Reproduced with permission [106]. Copyright 2023, Springer Nature.

### 3.2 Free Energy‐Mediated Hairpin Self‐Assembly

Self‐assembly reactions represent a distinctive class of NA amplification techniques that operate independently of NA polymerases, deconjugating enzymes, or comparable components. This group includes the pioneering hybridization chain reaction (HCR) [107], conceived by Dirks and Pierce, alongside the more advanced catalytic hairpin assembly (CHA) [108]. Notably, the HCR method integrates the target molecule into the expanding double helix, whereas in the CHA method, the target molecule functions primarily as a catalyst. Ying and Li et al. leveraged the chromogenic properties of gold nanoparticles (AuNPs) to label hairpins in HCR and CHA reactions, achieving ultra‐low limits of detection (LOD) for *Salmonella* genes (1.76 pmol L^−1^) and miRNAs (100 fmol L^−1^) (Figure 4C) [104, 109]. These methods have been adapted for use with fluorescent substances and surface‐enhanced Raman scattering (SERS) labels [110, 111, 112]. Recently, an isothermal amplification technique called “tile self‐assembly” has gained prominence in the field of NAT. Unlike the fixed hairpin structures in HCR and CHA, tile self‐assembly allows for the construction of 2D or 3D shapes using various DNA tiles with specific shapes and bonding ends, offering high customizability and programmability [113]. Brannetti et al. designed DNA tubular structures using tiles decorated with reporter tags and recognition, which can serve as scaffolds for different recognition elements and fluorescent dyes [105]. These structures have been integrated with LFAs, demonstrating multiplexing capabilities (Figure 4D). This contribution represents a significant improvement of paper‐based NAT, extending its applicability through DNA self‐assembly technology. Similarly, Wang et al. applied self‐assembly in LFA design with four functional oligonucleotides that bind to signaling molecules, achieving an LOD of 0.36 pM for TK1 mRNA detection (Figure 4E) [106].

Enzyme‐free methods offer greater flexibility and cost advantages but face challenges such as spatial constraints within the paper's pore structure and altered reaction kinetics due to the elongation of NA chains. These issues can lead to extended reaction times to reach equilibrium and the risk of non‐specifically adherence of long‐chain NAs to paper, potentially causing false positives or negatives [95]. Possible solutions to overcome these challenges include the use of paper with uniform and larger pores to create spatial conditions for the extension of long‐chain NAs within the paper matrix; employing microfluidic technology or printing techniques to control liquid flow and reaction areas, thereby increasing the local concentration of reactants and reaction efficiency; determining the optimal incubation time through experimentation to ensure sufficient amplification time while avoiding non‐specific reactions caused by excessive amplification.

### 3.3 Toehold Switch‐Mediated Gene Expression

The Toehold switch, a quintessential synthetic biology tool, amplifies signals in paper‐based assays by harnessing the programmability and modularity of synthetic biology, while retaining the simplicity and affordability of paper platforms [114]. Unlike traditional amplification methods, the Toehold switch relies on a stable secondary structure that prevents ribosome binding in the absence of a target. Upon target binding, the secondary structure is disrupted, allowing ribosomes to access the reporter gene and initiate translation, leading to a detectable signal (Figure 5) [115]. The switch's versatility with various reporters, including colorimetric and fluorescent proteins, extends its utility in paper‐based diagnostics. Cao et al.’s study exemplifies this by integrating the *LacZ* reporter gene with NASBA, converting target RNA presence into a sensitive, real‐time colorimetric process, with exceptional sensitivity in detecting respiratory syncytial viruses A and B at 52 and 91 aM LODs, respectively [80]. Karlikow et al. further enhanced detection sensitivity by incorporating NASBA pre‐amplification and magnetic bead‐based target enrichment, achieving LODs of 2 aM for Zika virus and 270 zM for chikungunya virus [116]. The growing interest in multiplexed paper‐based assays is met by the Toehold switch's ability to design orthogonal switches activated by distinct target sequences without cross‐talk. The versatility of the system has been enhanced by the integration of bioluminescent reporter genes, notably NanoLuc, and green fluorescent protein (GFP) genes [117, 118]. It is widely used in the detection of gut microbiota and other infectious diseases [119]. Nonetheless, the stability of the Toehold switch in non‐refrigerated environments presents a challenge, as RNA molecules, being more prone to degradation than DNA, could impact long‐term storage and transportation [120].

**FIGURE 5:**
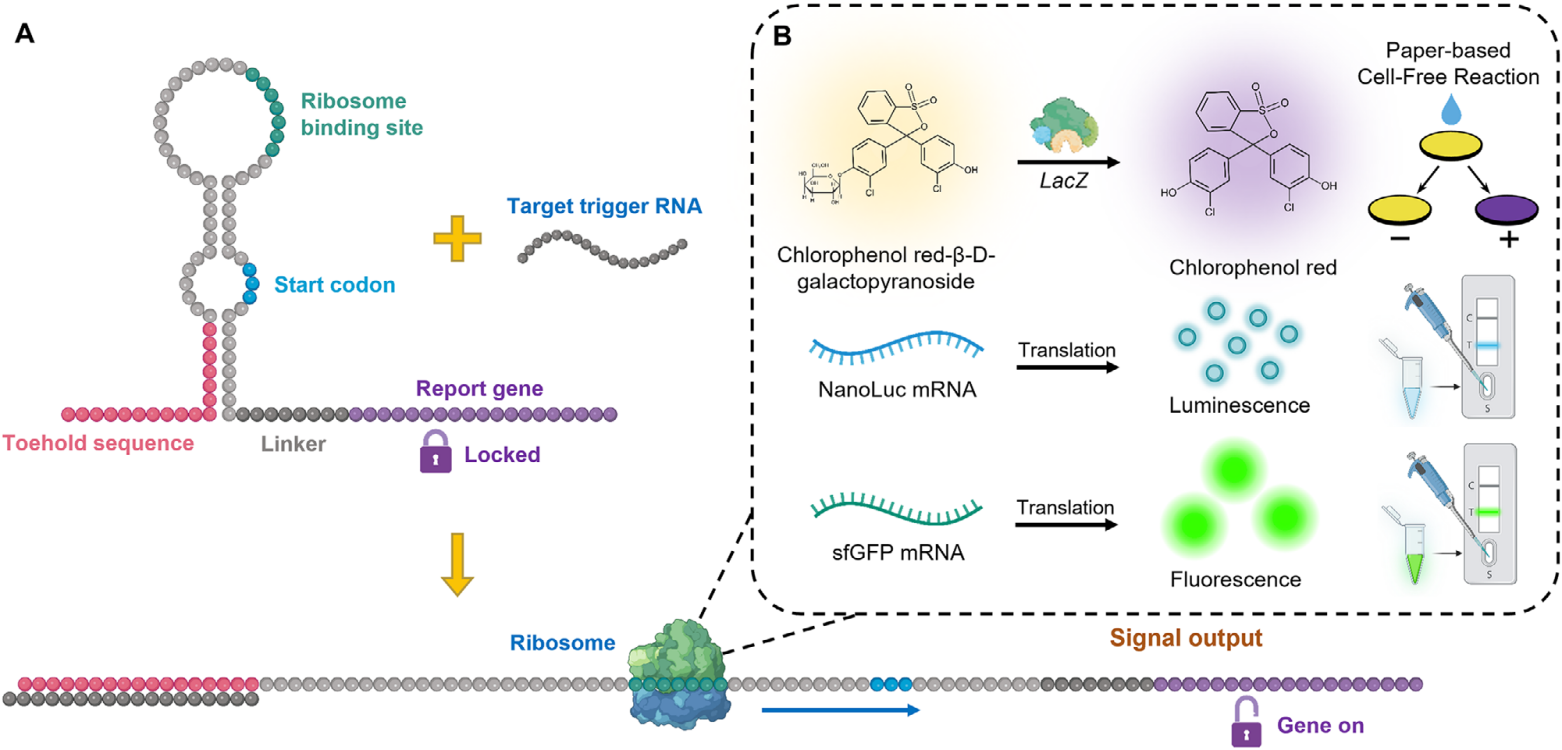
Toehold switch‐based diagnostics. (A) Regulatory mechanism of the toehold switch. In the absence of trigger RNA, RBS and start codon are hidden in the hairpin loop structure and inaccessible to the ribosome. (B) In the presence of trigger RNA, the RBS and start codon are released to translate reporter genes, resulting in expression that acts as a signal detectable by the naked eye or specific instruments.

### 3.4 Amplification‐Free Detection Techniques

The integration of NA amplification in PADs significantly enhanced analytical performance, yet it poses challenges such as extended analysis times, the need for additional devices, and the risk of errors like amplification bias and cross‐contamination [121]. The CRISPR‐Cas system has been a significant innovation in molecular biology, offering programmable and specific NA recognition capabilities that have revolutionized the development of highly sensitive and specific assays, but typically requires an additional amplification step to improve analytical performance [95]. To address this limitation, researchers are engineering the CRISPR‐Cas system to boost its native efficiency and hopefully, achieve highly sensitive detection in combination with PADs under amplification‐free conditions. This is being pursued through two main strategies: enhancing the targeting ability and enzymatic efficiency. One innovative approach is the use of multiple crRNAs to increase Cas13a activation, as shown by Fozouni's team, which leverages collective cleavage activity to reduce assay time and enhance the LOD for targets like SARS‐CoV‐2 [122]. This method allows for the detection of 100 copies mL^−1^ of pre‐isolated SARS‐CoV‐2 RNA on a mobile device within 30 min (Figure 6A). However, there is still a need for faster and more sensitive one‐pot detection chemistries for widespread POC diagnostics [123]. An alternative approach to improving enzyme efficiency involves using multiple CRISPR nucleases for direct NA sensing and rapid signal generation. Liu et al. combined RNA‐guided Cas13 and Csm6 with stable activators to develop a one‐step assay that enables the detection of approximately 30 molecules µL^−1^ of RNA within 20 min (Figure 6B) [124]. These strategies provide a direction for developing highly sensitive paper‐based assays without amplification, in conjunction with advanced CRISPR technologies. As demonstrated by Long et al., who manipulated the crRNA design by extending the 3′‐ or 5′‐ends with various lengths of ssDNA, ssRNA, and phosphorothioate ssDNA [125]. Their work revealed a self‐catalytic phenomenon and a remarkable increase in LbCas12a‐mediated collateral cleavage activity, enhancing detection up to 3.5 times compared to the wild‐type crRNA. This improvement also resulted in a significant boost in the specificity of target recognition, reaching detection limits in the femtomolar range without necessitating any preliminary amplification steps. These strategic advancements in CRISPR‐Cas system engineering underscore the potential for developing highly sensitive PADs that operate under amplification‐free conditions.

**FIGURE 6:**
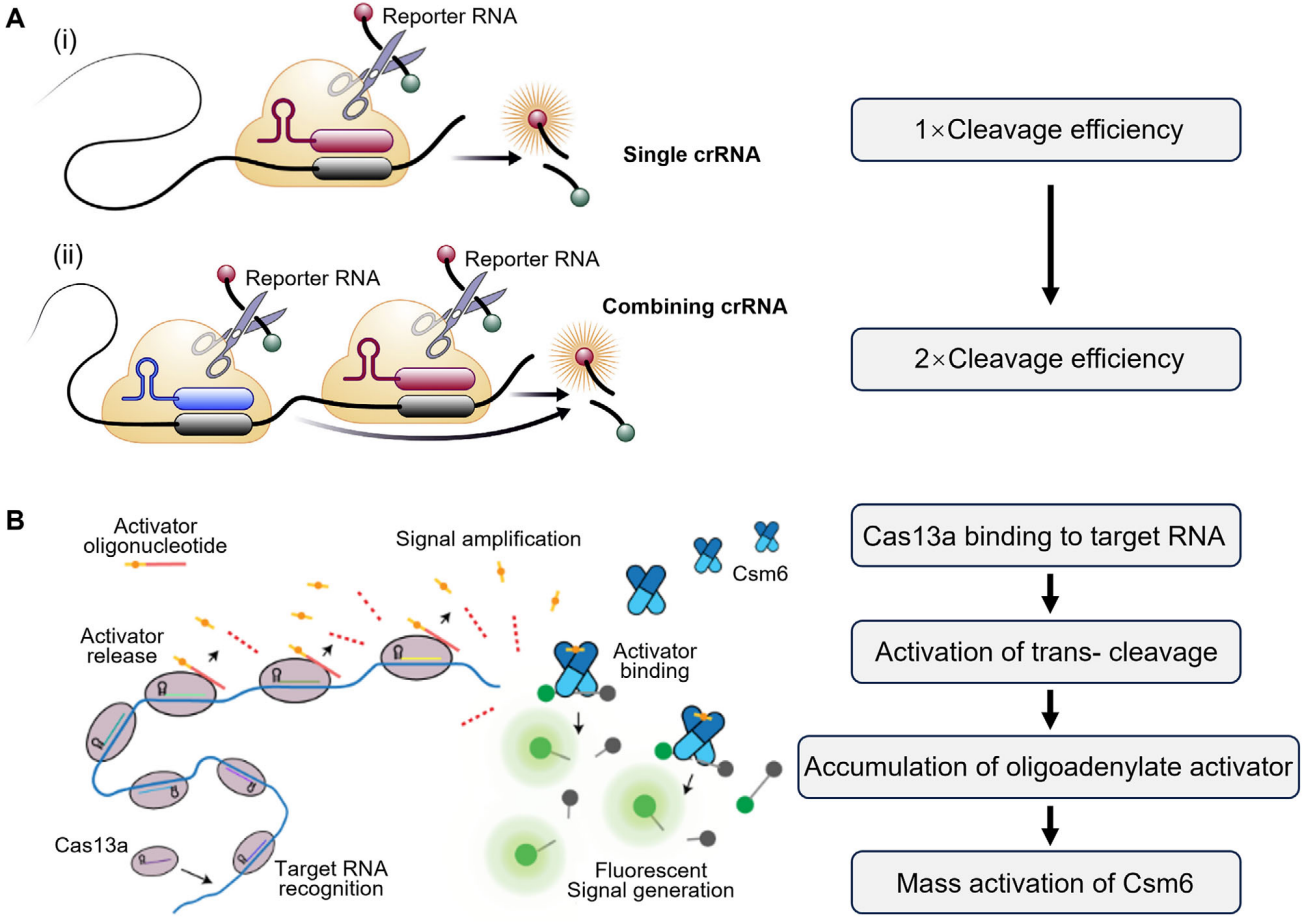
Improved CRISPR technology for amplification‐free detection. (A) Combining crRNAs improves the sensitivity of Cas13a. Reproduced with permission [122]. Copyright 2021, Elsevier. (i) Single crRNA‐mediated Cas13a activation and fluorescent reporter group cleavage. (ii) Combining multiple crRNAs to increase Cas13a activation. (B) Tandem use of Cas13 and Csm6 provides direct RNA sensing and rapid signal generation. Reproduced with permission [124]. Copyright 2021, Springer Nature.

The exploration of electrochemical paper‐based analytical devices (ePADs) further expands the repertoire of amplification‐free NAT methods compatible with PADs. These assays function through the measurement of current response, which is inherently highly sensitive. Appan Roychoudhury's team developed ePADs based on electrochemical impedance spectroscopy, employing multivalent binding technology [126]. This method increases the frequency of interactions between probes and targets and has successfully detected SARS‐CoV‐2 RNA without amplification, achieving a LOD of 303 aM. Koo et al. used alterable alternating current electrohydrodynamic forces to enhance probe‐target hybridization before direct native RNA target detection, without target amplification or surface functionalization [127]. These methods facilitate the development of ePADs for amplification‐free NAT by boosting the current response through increased electrochemical probe‐target hybridization, thus meeting the sensitivity thresholds required for detection. However, low stability and interference from background noise remain one of the major limitations in the development of ePADs. To overcome these, researchers are investigating the potential of stabilizing agents, such as polymers and protective coatings, to maintain the structural integrity of the electrochemically active components on the paper surface [128, 129, 130]. The application of statistical algorithms and machine learning techniques to raw electrochemical data enables researchers to distinguish real signals from background fluctuations, thereby enhancing the reliability of detection results.

## 4 Signal Output for Paper‐Based NAT

Recent advancements in nanotechnology have driven changes in NA labeling and signaling methods in paper‐based NAT. Nanomaterials play a pivotal role in enhancing NA detection performance by amplifying signal strength, improving sensitivity, and enabling rapid and accurate readouts. Their unique electronic, optical, catalytic, and structural properties contribute to substantial improvements in NA analytical capabilities, promoting the development of highly sensitive, robust, and user‐friendly paper‐based NAT systems. Materials such as carbon nanotubes, quantum dots (QDs), and nanozymes are frequently employed to reinforce NA signal strength, enhance stability, and facilitate quantitative detection. Here, we highlight recent advances in various nanomaterials with excellent properties for NA labeling, with a special focus on noble metal nanomaterials, carbon nanomaterials, fluorescent materials, and nanozymes. In Table 2, we summarize the various analytical methods involving these materials.

**TABLE 2:** Analytical technologies and representative nanomaterials for paper‐based NAT platforms.

Technology	Nanomaterials	Specificities	Advantages	Limitations
Colorimetry	AuNPs or AgNPs aggregation; nanozymes	Nanoparticle aggregate colorimetric intensity depends on size and shape; nanozymes exhibit higher catalytic activities and false positive rates.	Naked‐eye detection; simple and rapid; low‐cost; no need for an expensive instrument; signals can be read by a smartphone or portable reader.	Limited sensitivity; vulnerable to environmental; color variation; subjective.
Raman spectra	AuNPs or AgNP‐based SERS tags	Nanoparticle shape significantly affects SERS activity; Ag oxidizes easily and degrades SERS activity.	Low LODs; label‐free detection; multi‐component analysis.	Vulnerable to background noise; expensive instrument; complicated operation; time‐consuming.
Fluorescence	QDs; CDs; UCNPs	QDs possess multi‐labeling capabilities; plasmonic fluor enhancement; high‐contrast imaging based on FRET and UCNPs.	Low LODs; multi‐detection capability; qualitative and quantitative analysis; ratiometric fluorescence detection is available.	Vulnerable to environmental conditions; requires additive‐free paper to improve signal‐to‐noise ratio; photobleaching.
Electrochemistry	Carbon nanotube; graphene	High electron mobility; fast and sensitive current response.	Low‐cost; easy to miniaturize and integrate; rapid; low LODs.	Electrode contamination and wear; electrode handling; standardization and calibration; low repeatability.

### 4.1 Precious Metal Nanomaterials

Precious metal materials, including Au and Ag, display distinctive nanoscale attributes, such as size‐ and surface‐dependent effects, which significantly enhance optical signal generation and amplification, making them pivotal for improving the sensitivity and reliability of paper‐based NAT platforms. These nanoparticles can be conjugated to DNA or RNA through electrostatic adsorption, covalent bonding (notably gold–sulfur bonds), or the biotin‐affinity system, making them suitable for signal amplification in PADs. AuNPs are particularly favored in PADs for colorimetric assays due to their strong surface plasmon resonance (SPR) [131]. The morphology of these nanoparticles is pivotal for PAD sensitivity. Li and Zhan et al. demonstrated that AuNPs with a size of around 100 nm achieve an optimal balance between molar extinction coefficient and reaction rate [132, 133]. Additionally, gold nanoflowers of the same size as spherical or rod‐shaped AuNPs have higher molar extinction coefficients and larger specific surface areas, leading to stronger colorimetric signals and higher NA or enzyme coupling densities. For example, in Zhang et al.’s study, gold–platinum nanoflowers significantly enhance NA detection by serving as both colored and catalytic labels, amplifying the signal in lateral flow assays. Their dual functionality allowed for the detection of miRNA‐21 at 0.3 pM, 200 times more sensitive than traditional methods, demonstrating their potential in ultrasensitive, on‐site NA biosensors for biomedical diagnostics [134]. While AgNPs have been used in paper‐based colorimetric detection, their standalone use is discouraged due to their weak contrast on white paper, especially at low NA target concentrations [135, 136]. However, reducing Ag^+^ in a silver enhancement solution to solid silver particles on the surface of gold particles under the action of a reducing agent can significantly enhance the detection sensitivity of NA, achieving a 100‐fold increase compared to using AuNPs alone [137].

Another important characteristic of gold and silver is their strong plasmonic activity, making them the most commonly used and strongest SERS‐active materials [138]. Paper‐based Raman spectroscopy detection based on the SERS effect typically exhibits superior detection performance compared to traditional LFA based on colloidal gold aggregation colorimetry [139, 140]. However, nanoparticles of the same material can exhibit vastly different SERS activity. Changing the shape of the nanoparticles can lead to more enhancement due to the presence of additional hotspots at the edges and corners [141]. Nanorods [142], nanospheres [143], nanostars [144], and nanoflowers [145] are commonly used for Raman tags. Typically, nanostars and nanoflowers are capable of producing strong localized electromagnetic field enhancements at their tips and grooves due to their unique sharp corners or dendritic structures, resulting in the formation of high‐density hotspots for superior NA detection performance [141, 146]. Jin‐Ha et al. developed a system that integrates activated CRISPR‐Cas12a technology with a Raman‐sensitive mechanism [147]. The system utilizes a substrate made of ssDNA‐immobilized, Raman probe‐functionalized AuNPs arranged on a GO/triangular gold nanoflower array. Using a CRISPR‐enhanced Raman‐sensitive approach, the team achieved a significant improvement in the detection sensitivity for multiple viral DNAs with an LOD of 1 aM, which is at least three orders of magnitude more sensitive than traditional SERS using nanospheres [148].

It is noteworthy that AgNPs have the strongest SERS activity, which significantly enhances the sensitivity of NA detection. However, AgNPs are prone to oxidation when exposed to air and can react with atmospheric sulfides, leading to a potential reduction in their SERS performance [149]. In comparison, AuNPs are more chemically stable, maintaining consistent performance over time. While they are more expensive, their stability makes them more suitable for long‐term use in enhancing the signal output in NA detection systems.

### 4.2 Fluorescent Nanomaterials

Fluorescent nanomaterials, particularly QDs and carbon dots (CDs), are extensively utilized in PADs for NA labeling due to their superior optical properties, such as larger Stokes shifts, enhanced photostability, and increased luminosity [150]. QDs stand out for their multimodal imaging capabilities. For instance, based on the photoelectric activity of quantum dots and the cascade sensitization effect of ZnO/CuInS2/Ag2Se, Hu et al. developed a paper‐based photoelectrochemical biosensor capable of the sensitive detection of miRNA‐141 with an LOD of 16.7 fM [151]. Su and co‐workers used the bright fluorescent labeling of the hairpin structures in the CHA reaction by QDs, achieving ultrasensitive detection of the hepatitis C virus RNA with an LOD 5000 times lower than that of conventional fluorescence‐based assays [152]. In addition, CDs also exhibit strong fluorescence and are used in PADs for the highly sensitive detection of cancer‐related miRNAs and viruses [153, 154].

SPR from metal nanostructures has been reported to significantly enhance fluorescence brightness [155, 156], thereby improving the sensitivity and performance of NA detection systems. When light irradiates a metal surface, it induces collective oscillation of free electrons, leading to SPR and a strong electromagnetic field near the surface, which amplifies the electric field component and light energy density [157]. This field amplifies the electric field component and light energy density, enhancing the signal output. By placing fluorescent substances near metallic nanomaterials, the fluorescence signals can be greatly amplified, leading to more sensitive detection of NAs [158]. Rohit Gupta et al. demonstrated that antibody‐conjugated fluorescent gold nanorods (AuNR) can render LFAs ultrasensitive, with fluorescence signals nearly 7000 times brighter than conventional fluorophores [156] (Figure 7). Based on this, Lip Ket Chin et al. developed a plasmonic sensing chip that integrates chemical fluorescence signal amplification with plasma‐enhanced fluorescence detection [155], enabling rapid, ultrasensitive multiplexed plasma sensing within 1 h with a sensitivity below pg mL^−1^. It is worth noting that the enhancement of this fluorescence effect is highly dependent on the type of metal material, particle size, shape, and the distance between the metal surface and the fluorescent substance [159, 160]. Overcoming these dependencies requires careful optimization to prevent signal quenching or diminished enhancement effects, ensuring the highest possible sensitivity and accuracy in NA detection.

**FIGURE 7:**
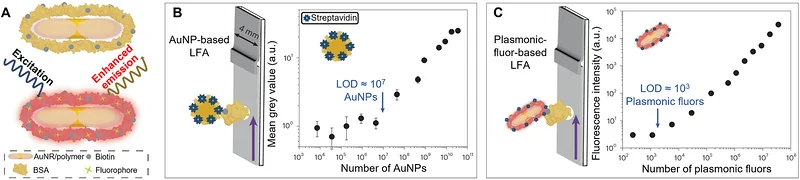
AuNPs and plasmonic fluors as nanolabels for LFA. (A) Schematic illustration of plasmonic fluor, colorimetric, and fluorescent bimodal nanolabeling was used in the LFA. (B) Mean gray values were measured from nitrocellulose membranes after exposure to varying concentrations of streptavidin‐conjugated AuNPs. (C) Fluorescence intensities were obtained from nitrocellulose membranes after exposure to different concentrations of streptavidin‐conjugated plasmonic fluors. Reproduced with permission [156]. Copyright 2023 Springer Nature.

Another significant application of fluorescent nanomaterials in PADs involves the use of the Förster resonance energy transfer (FRET) effect and the employment of upconversion nanoparticles (UCNPs) for high‐contrast imaging. These methodologies are particularly effective in enhancing the sensitivity of NA detection by reducing fluorescence interference in samples, which is crucial when detecting low target concentrations [161, 162]. The high quantum yield of QDs makes them ideal candidates as donors in FRET systems, further amplifying signal output and improving detection performance. Omair Noor et al. highlighted this advantage through a multiplexed solid‐phase NA hybridization assay on a paper platform, utilizing QD‐Cy3 and QD‐Alexa Fluor 647 FRET pairs for high‐contrast imaging [163]. One of the advantages of FRET‐based transduction is that it requires no additional washing step to remove unhybridized oligonucleotide sequences, thus simplifying the procedure. Additionally, introducing an internal standard allows for a self‐calibration mechanism through differences in fluorescence intensity at different wavelengths, increasing the upper limit of NA measurement sensitivity and dynamic range [154, 164]. UCNP plays a crucial role in enhancing NA detection by reducing background fluorescence interference, thus improving the signal output. These nanoparticles, doped with lanthanides, exhibit prolonged luminescence lifetimes (µs–ms), which allows for effective separation of background fluorescence using time‐resolved fluorescence techniques [165]. This characteristic facilitates the separation of background fluorescence through time‐resolved fluorescence techniques before assessing the near‐infrared (NIR) probe's fluorescence [166]. Furthermore, the excitation of the NIR probe by near‐infrared light, coupled with the non‐fluorescent nature of most substances in this sub‐band, results in imagery with high signal‐to‐noise ratios [167].

### 4.3 Enzyme‐Mimicking Nanomaterials

Enzyme‐mimicking nanomaterials, or nanozymes, provide cost‐effective and stable alternatives to traditional enzymes, making them ideal for POC applications, particularly in enhancing NA detection performance. Nanozymes derived from materials such as metal oxides, carbon‐based nanomaterials, and metal‐organic frameworks (MOFs) can significantly amplify the signal output in PADs. These nanozymes can be integrated into PAD systems through various strategies, including physical adsorption, covalent bonding, and biotin‐streptavidin systems, further enhancing the sensitivity and reliability of NA detection [168, 169, 170]. Marta Broto et al. developed a CRISPR‐Cas‐based NA assay using Pt@Au nanozyme‐linked immunosorbent assay, which enables the quantitative and colorimetric detection of Cas13‐mediated RNA [171] (Figure 8A). This approach underscores the high catalytic activity of nanozymes at room temperature, allowing for the sensitive detection of multiple non‐coding RNAs in LFAs with a LOD of 12.5 pM. However, the functionalization of nanozymes with biorecognition elements may reduce catalytic rate efficiency due to steric hindrance, necessitating careful evaluation of functionalization impact. On the other hand, Li et al. designed a highly selective and disposable paper‐based photoelectrochemical (PEC) sensor for miRNA‐141 detection, based on the simple self‐assembly of a target‐triggerable DNA motor and a nanozyme‐catalyzed multistage biocatalytic precipitation reaction [172], achieving highly sensitive detection of miRNA‐141 with a LOD of 0.6 fM (Figure 8B).

**FIGURE 8:**
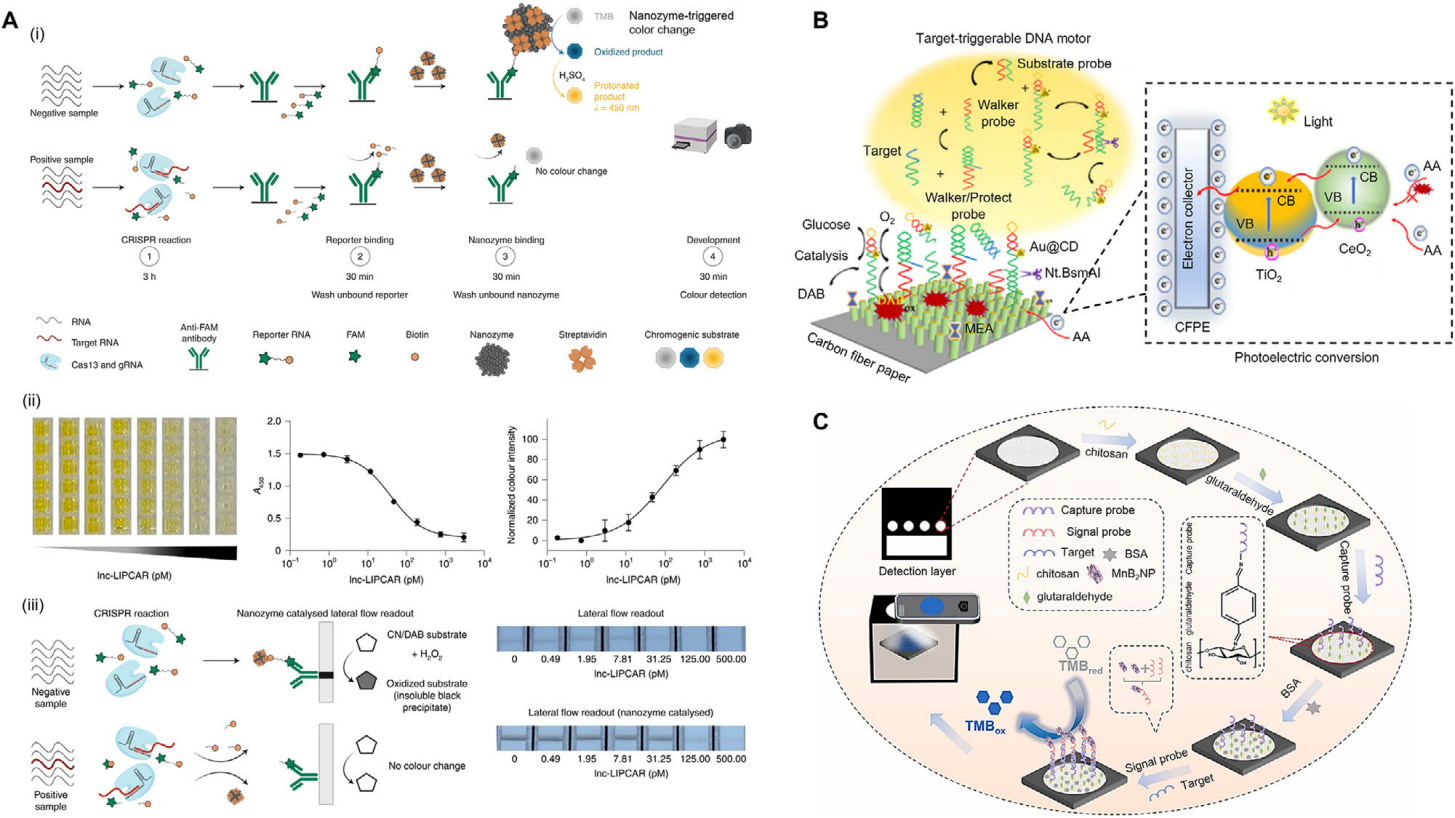
Nanozymes for high‐performance paper‐based NA sensing. (A) CrisprZyme assay scheme. Reproduced with permission [171]. Copyright 2022, Springer Nature. (i) Schematic of the combination of a Cas‐based reaction with a nanozyme‐amplified LFA. (ii) CrisprZyme detects synthetic RNA down to picomolar concentration. (iii) Schematic of the combination of a Cas‐based reaction with a nanozyme‐amplified LFA. (B) PEC sensor based on TiO_2_/CeO_2_ heterojunction and dual‐enzyme‐engineered DNA walker. Reproduced with permission [172]. Copyright 2020, American Chemical Society. (C) Flowchart of ctDNA detection by portable smartphone based on MnB_2_ nanozyme and PAD. Reproduced with permission [173]. Copyright 2024, Elsevier B.V.

As research in nanozymes progresses, there is an increasing focus on discovering new materials and engineering synthesis strategies to enhance catalytic activity and multi‐functionality, which directly contribute to the improvement of NA detection performance. By employing biological templates, self‐assembly techniques, and computer‐aided design, nanozymes can be engineered with highly refined structures and functionalities that are tailored for specific detection tasks. These optimized nanozyme structures can respond to changes in the environment (e.g., pH, temperature, or the presence of specific biomolecules), significantly enhancing the signal output and accuracy of NA assays, as well as improving the performance of therapeutics [174, 175, 176]. Yang et al. developed a portable POCT platform for circulating tumor DNA (ctDNA) detection using MnB_2_ nanozyme and a PAD, with a smartphone app for signal analysis (Figure 8C) [173]. The method was successfully applied to detect ctDNA in tumor cell lysates and peripheral blood samples from tumor‐bearing mice, with results corroborated by the standard qPCR method, confirming the reliability of the POCT device for ctDNA detection. Nevertheless, the high catalytic activity of nanozymes, while beneficial for enhancing NA detection performance, also presents challenges in PAD design, particularly in minimizing non‐specific binding that could lead to false positives [177, 178]. To overcome this, future development of PADs should focus on surface modification to prevent aggregation, optimizing detection conditions (such as pH, temperature, and ionic strength) that could influence nanozyme activity and stability, and carefully selecting appropriate blocking agents. Additionally, the design of dual detection systems incorporating two or more nanozymes with complementary specificities could further enhance the signal output and improve the accuracy of NA detection.

### 4.4 Carbon Nanomaterials

Carbon nanomaterials are commonly used in the construction of ePADs, such as, carbon nanotubes and graphene, which are valued for their exceptional electrical conductivity and abundance of active surface sites. These nanomaterials significantly enhance NA detection performance by improving electron mobility and electrochemical stability, which directly contribute to more sensitive and reliable signal output. Compared to standard carbon, gold, or silver electrodes, carbon‐based nanomaterials provide superior performance [179]. Graphene‐based electrodes, for example, have demonstrated significant improvements in these areas, making them ideal for electrochemical sensing in NA detection applications. Antonia Perju and colleagues employed a graphene carbon nanofiber prepared by laser induction to illustrate the potential of graphene‐based electrodes in ePADs for the detection of *Cryptosporidium* DNA, achieving an exceptional LOD of 137 pM [55]. However, challenges such as insufficient detection flux in lateral flow channels necessitate device design optimization. Li and colleagues addressed this by integrating GO‐hybridized multi‐walled carbon nanotube (MWCNT) nanocircuits into a paper heater, achieving an LOD of 25 copies mL^−1^ for the SARS‐CoV‐2 N gene, which is 40 times more sensitive than conventional PCR [29]. Despite these advancements, the quest for high sensitivity in ePADs must be balanced against the potential loss of specificity, which can arise from electrode surface contamination, wear, and non‐specific adsorption [180, 181]. Strategies to mitigate these issues involve the self‐assembly of methylene blue‐modified capture probes on the working electrode, serving as an internal reference element. Additionally, the design of multipath self‐cleaning labels allows for time‐programmable cleaning of the working electrode, further enhancing the specificity and reliability of ePADs [182].

Carbon nanomaterials offer a versatile avenue for enhancing the performance of ePADs in NAT. Their integration into PADs not only boosts sensitivity but also greatly contributes to the enhancement of signal output, addressing key challenges such as specificity and device stability. These nanomaterials enable more precise NA detection, making ePADs more reliable for POC diagnostics. Ongoing research in this domain is likely to yield further breakthroughs, making ePADs an increasingly attractive option for POC diagnostics and global health monitoring.

## 5 Conclusion and Perspectives

The paper‐based assay has advanced significantly since its inception in the early 21st century and has become a crucial component of the POC field. Research has yielded substantial improvements in detection sensitivity and functional integration in fast NA detection [2, 183]. Paper‐based POCT devices leverage several key advantages over traditional POCT devices, including their portability, disposability, and the potential for low‐cost mass production [184, 185, 186]. Moreover, the simplicity of use and the minimal infrastructure required for these devices make them highly accessible in resource‐limited settings [187]. Despite initial challenges such as low sensitivity and specificity and the complexity of integrating a comprehensive “sample‐in, answer‐out” system, recent advancements have begun to surmount these obstacles. Moreover, the advancement of portable signal analysis instruments and the integration of machine learning algorithms are augmenting the quantitative analytical capabilities of paper‐based microfluidics in NA detection, steadily progressing toward intelligence and universality. It is anticipated that PADs for NAT will achieve continuous advancements in the following areas in the future.

### 5.1 Visualization

PADs with strong on‐site testing capabilities must provide clear, intuitive results quickly and be user‐friendly. The avant‐garde in this domain is bifurcated into two principal thrusts: First of all, the integration of ultra‐sensitive nanomaterials and the employment of signal‐amplification labeling techniques. These innovations are replacing traditional AuNPs, thereby augmenting the perceptibility of visual signals [188, 189, 190, 191]. Then, there is a concerted effort to mine into and harness high‐sensitivity NA detection technologies based on existing POCT frameworks, such as PTS [192]. This strategy, which repurposes existing POCT devices for detection, avoids the most time‐consuming processes associated with device design, optimization, and manufacturing [95, 193]. This not only provides a more efficient solution but also signals a trend towards a future where unassisted self‐testing becomes increasingly accessible and effective, particularly at the onset of large‐scale infectious disease outbreaks.

### 5.2 Amplification‐Free Detection

NA detection typically requires several sample‐pretreatment steps, including reagent mixing, cell lysis, and NA purification and concentration, in contrast to the more straightforward detection of protein macromolecules, such as that used in pregnancy strips. Despite the pretreatment enhancing the concentration of target molecules in paper‐based detection, capturing them within a constrained timeframe remains challenging. It is essential to understand that amplification steps are fundamental to paper‐based NAT processes. Although isothermal amplification technology supersedes traditional PCR technology, enhancing both sensitivity and practicality. However, the time needed for rapid screening of large populations remains a critical consideration. The primary challenge is converting trace amounts of NA targets in complex samples into quantifiable detection signals [194, 195]. To overcome this, employing microfluidic and kinetic designs that increase the collision probability between trace NAs and probe molecules is a promising approach.

### 5.3 Enhancement of Applicability Based on Digital Technology

The substantial increase in computing power, along with the widespread adoption of mobile‐health approaches, significantly advanced the development of portable analyzers and digital analysis techniques in biomedical testing [196, 197, 198, 199]. A notable example of this advancement is the interpretation of test results via smartphones [200, 201]. By leveraging advanced computing and imaging technologies, this method utilizes the smartphone's camera or modified optics, such as filters, to directly analyze signal areas [202, 203]. This approach provides personalized diagnostic insights with enhanced sensitivity, allowing for the analysis of complex data beyond the capabilities of the naked eye. Additionally, the integration of portable devices such as fluorescence analyzers, IoT, and so on has facilitated the miniaturization of bulky instruments, making them compatible with PADs for POC applications, particularly in resource‐limited settings [204, 205, 206]. The ability to perform complex analyses with simple devices in various settings highlights the importance of further integrating digital technologies into biomedical testing to meet the challenges of the future.

### 5.4 Commercialization

PADs for NAT possess substantial potential in the realm of decentralized diagnostics [207, 208]. Currently, the market has witnessed the emergence of various cost‐effective PADs designed to streamline the fundamental steps of NAT. However, the commercialization of these devices has proceeded at a slower pace compared to their research and development. While commercial NAT solutions like real‐time PCR and next‐generation sequencing have become the market standard due to their reliability and performance, PADs for NAT are gaining commercial traction, particularly in LFAs and qualitative tests for diseases such as influenza and COVID‐19. Despite this, the shift from research prototypes to market‐ready products has been gradual, facing challenges in manufacturing scale‐up, regulatory approval, and matching the performance of conventional methods. The market success of PADs hinges on their ability to satisfy the rigorous demands of clinical diagnostics while providing the advantages of decentralized testing. Advancing PADs for NAT through innovative material science, molecular biology, and digital technology will be crucial for their future commercial success [209]. With the escalating global need for rapid, accessible, and cost‐effective diagnostic tools, especially against the backdrop of emerging infectious diseases and an aging population, the commercialization of PADs for NAT is poised for growth.

In conclusion, paper‐based NA analysis technology integrating multiple amplification strategies has been widely used in the quantitative detection of various pathogenic microorganisms and disease markers and has shown great advantages and prospects in portable, multi‐target, and rapid detection. The integration of diverse nanomaterials, analytical chemistry, molecular biology research, and digital analysis methodologies will propel the advancement of paper‐based NAT. The process will entail the miniaturization of electronic components and the attainment of significant functional integration, facilitating real‐time data analysis and the wireless transmission of test results to healthcare professionals. We expect the effective large‐scale implementation of digital paper‐based detection sensing for NAT from laboratory settings to clinical practice.

## Author Contributions

**Hong Zhong**: conceptualization, funding acquisition, supervision, writing – review and editing. **Yi Yang**: writing – original draft, writing – review and editing. **Heng Li**: writing – original draft, writing – review and editing. **Xiaolin Hu**: investigation. **Hui Xiao**: investigation, data curation. **Dou Wang**: editing. **Sergio Benardini**: editing. **Wei Gu**: supervision, funding acquisition, writing – review and editing. **Yang Luo**: conceptualization, funding acquisition, supervision, writing – review and editing.

## Conflicts of Interest

The authors declare no conflicts of interest.
